# Key extracellular proteins and TF-miRNA co-regulatory network in diabetic foot ulcer: Bioinformatics and experimental insights

**DOI:** 10.1371/journal.pone.0307205

**Published:** 2024-07-22

**Authors:** Guanlin Lin, Ximing Liu

**Affiliations:** 1 Department of Orthopaedic Surgery, General Hospital of Central Theater Command, Wuhan, China; 2 College of Acupuncture and Orthopaedic, Hubei University of Chinese Medicine, Wuhan, China; 3 The First Affiliated Hospital of Xiamen University, Xiamen, China; 4 The First School of Clinical Medicine, Southern Medical University, Guangzhou, China; Jashore University of Science and Technology, BANGLADESH

## Abstract

**Background:**

Diabetic foot ulcers (DFUs), a serious complication of diabetes, are associated with abnormal extracellular protein (EP) metabolism. The identification of key EPs and their regulatory networks is crucial for the understanding of DFU formation and development of effective treatments. In this study, a large-scale bioinformatics analysis was conducted to identify potential therapeutic targets and experimental validation was performed to ensure the reliability and biological relevance of the findings.

**Methods:**

Due to the comprehensive profiling of DFU samples provided by the GSE80178 dataset, we initially selected it to derive differentially expressed genes (DEGs) associated with DFU. Subsequently, utilizing the UniProt database and annotated EP list from the Human Protein Atlas annotation database, we screened for extracellular protein–related differentially expressed genes (EP-DEGs) due to their crucial role in the pathogenesis and healing of DFU. We examined EP-DEG pathway enrichment and protein-protein interaction networks, analyzed paired full-thickness skin tissue samples from 24 patients with DFUs and healthy controls, and performed polymerase chain reaction (PCR) experiments to validate candidate genes. Ultimately, we constructed a transcription factor (TF)-microRNA (miRNA)–hub gene co-regulatory network to explore upstream and downstream regulatory connections based on validated DEGs.

**Results:**

Four crucial candidate genes (FMOD, LUM, VCAN, and S100A12) were identified and verified via PCR analysis. The TF-miRNA-hub EP-DEG regulatory network contained the pivotal TFs TRIM28 and STAT3 and the miRNAs hsa-mir-20a-5p, hsa-miR-21, and hsa-miR-203.

**Conclusion:**

The findings of this study advance our understanding of the pathology of DFU by defining key roles of specific EPs and elucidating a comprehensive regulatory network. These insights pave the way for novel approaches to improve DFU treatment outcomes.

## Introduction

Diabetes imposes a considerable global public health burden, accounting for 80% of non-communicable disease-related deaths, including those due to cancer and respiratory and cardiovascular pathologies [1]. Contemporary studies forecast that this burden will continue to increase. The yearly incidence of types 1 and 2 diabetes increased from 1.8% in 2002 to 4.8% in 2012 [2], and the prevalence of diabetes increased by about 30.6% from 2005 to 2015 [2]. The estimated global population of diabetics is projected to surpass 570 million by 2025 [3]. Diabetes is defined by chronically elevated blood glucose levels, causing vascular, neural, and immune system damage. Hyperglycemia suppresses the cell growth and proliferation that are crucial for wound maturation, thereby impeding tissue repair [4]. Diabetic foot ulcers (DFUs) are a common chronic diabetic complication. Approximately 15% of patients develop DFUs (~6.3% worldwide prevalence) [5], and 85% of these cases necessitate amputation; the 5-year DFU survival rate is only 50.9% [6]. Diverse methods of DFU prevention and management exist, but they are insufficiently effective. The identification and understanding of key genetic targets and regulatory networks involved in the pathogenesis of DFU are crucial to develop more effective therapeutic strategies.

Extracellular proteins (EPs) are structural proteins that cells produce and secrete outside of cell membranes into the extracellular space. The class of EPs is broad, encompassing extracellular signaling molecules, enzymes, hormones, and other elements that participate in various biological processes [7]. EPs play essential biological roles in cell-to-cell signaling, extracellular matrix (ECM) construction and maintenance, immune regulation, and the regulation of cell proliferation, migration, differentiation, apoptosis, and ECM synthesis and degradation. Structural proteins such as fibronectin, elastin, and fibulin are EPs located in the ECM. The abnormal expression and dysfunction of EPs may lead to chronic non-healing of wounds [8], as EPs play decisive roles in wound repair and healing processes such as cell adhesion, migration, and signaling and ECM remodeling.

Diabetes is a metabolic disorder whose pathogenesis involves multiple factors, such as vascular damage, nerve fiber injury, immune dysfunction, and infection [9]. The increased release of EPs and signaling molecules can affect the foot vasculature and immune system, thereby increasing the risk of DFU development [10, 11]. Diabetic patients also experience high levels of inflammation and oxidative stress, which can affect the synthesis and degradation of EPs, subsequently influencing tissue repair and ulcer healing [12]. Thus, the targeting of key EPs holds promise as a potential approach to DFU treatment.

The Gene Expression Omnibus (GEO; https://www.ncbi.nlm.nih.gov/geo/) is a public functional genomics data repository managed by the National Center for Biotechnology Information, which is part of the United States National Library of Medicine. Its focus is to store, integrate, analyze, and share data from biological experiments, primarily gene expression and high-throughput data [13]. The GEO was created to facilitate global advancements and collaboration in the life sciences via the provision of a user-friendly, open, and transparent data resource. The data available in the GEO provide crucial information on gene expression and function that researchers can compare and analyze to draw valuable insights.

This study was conducted to explore the involvement of key EPs in the pathogenesis of DFUs and to identify potential biomarkers and therapeutic targets. To achieve this goal, we identified differentially expressed genes (DEGs) in DFU skin tissues using GSE80178 microarray gene expression profiles. We focused on DEGs encoding EPs, analyzing their biological functions and pathways, and constructed a protein-protein interaction (PPI) network to identify core EP genes (hub EP-DEGs). Validation was performed using skin tissue samples from patients with DFUs and healthy individuals. Additionally, we developed a transcription factor (TF)-microRNA (miRNA) co-regulatory network to understand the regulatory relationships of these hub EP-DEGs. Our findings provide new insights and a theoretical basis for the exploration of DFU mechanisms and treatment.

## Materials and methods

### Data collection and processing

**Fig 1:**
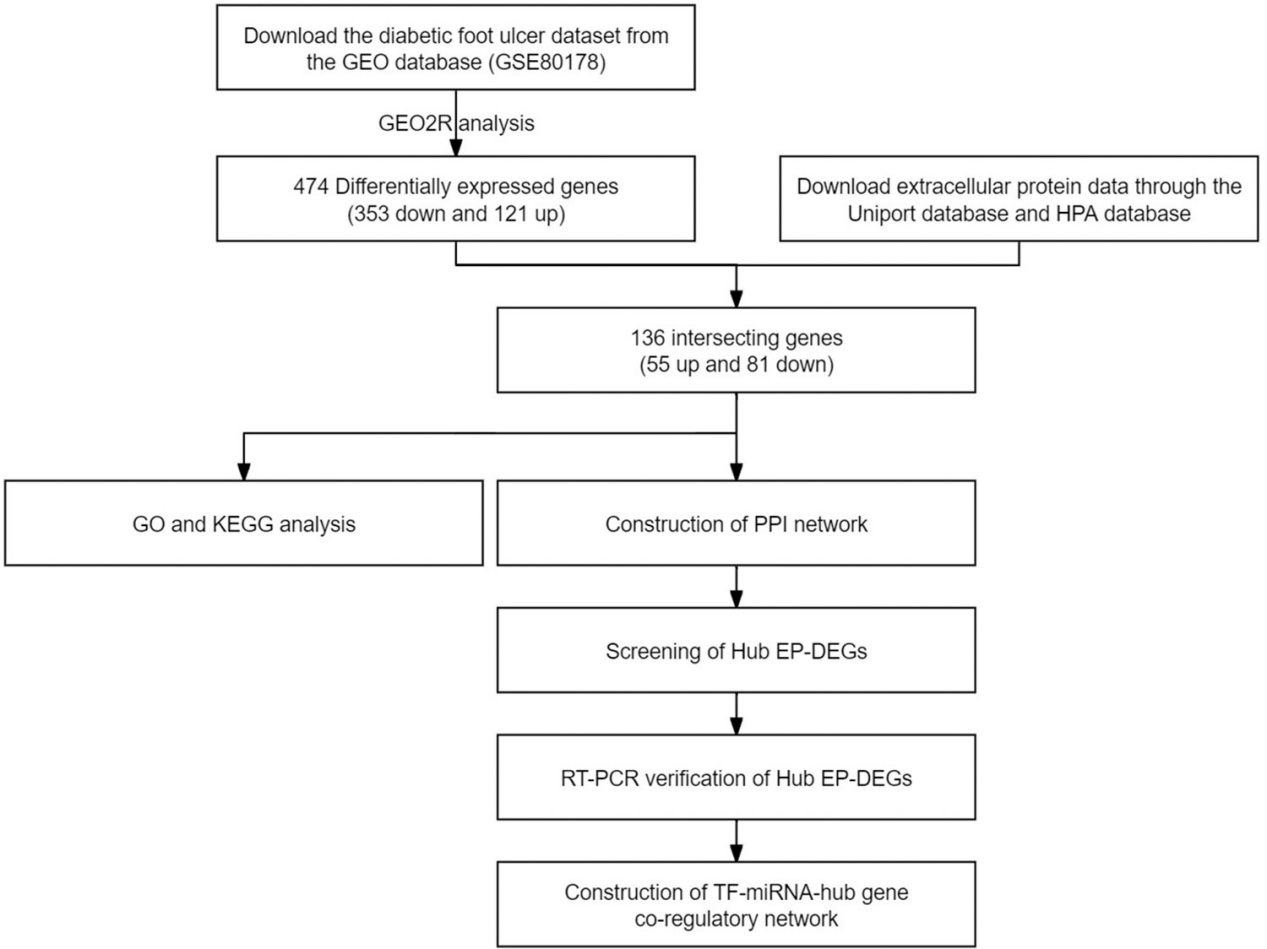
Research flow chart.

Fig 1 shows the flow of this study. The GSE80178 dataset from the GEO database [13] specifically focuses on full-thickness skin tissue samples from patients with DFU and non-diabetic controls, aligning well with our research objectives. From this dataset, we obtained gene expression data for six DFU patients and three non-diabetic controls (S1 Table). For the preliminary analysis of differential gene expression, we utilized the GEO2R online tool (available in the GEO database).

### Screening for DEGs

In the differential expression analysis, we set the thresholds of |log_2_ fold change| > 2 and *p* < 0.05. Log_2_ fold changes > 2 were taken to represent upregulated genes, and those <–2 were taken to represent downregulated genes. We used the ggplot2 (version 3.3.3) and ComplexHeatmap (version 2.2.0) packages to generate heatmaps and volcano plots to visualize expression patterns and identify differential expressions among the samples. After DEG filtering, we downloaded lists of EP genes from the UniProt database (https://www.uniprot.org/) [14] and the Human Protein Atlas (HPA) annotation database (https://www.proteinatlas.org/) [15], and identified intersections of these genes with the DEGs. The results were used to select EP-DEGs. We used the online tool available at https://bioinfogp.cnb.csic.es/tools/venny/index.html to generate a Venn diagram illustrating the intersection of protein targets.

### Analysis of EP-DEG functional enrichment

We conducted an enrichment analysis using the GO and KEGG databases to identify the biological processes and pathways associated with EP-DEGs. The GO database [16] is a standardized evolutionary biology database used for gene and non-coding RNA annotation and function and expression analyses. It integrates various aspects of gene annotations, such as biological processes (BPs), molecular functions (MFs), and cellular components (CCs), into a hierarchical structure. The KEGG bioinformatics database [17] provides multidimensional data, including that on gene annotation, pathway mapping, and relationship networks, covering biological aspects such as cellular processes, tissue development, metabolic pathways, and signal transduction. The analysis and visualization of the results were performed using the clusterProfiler packages in R (version 4.2.1), with the significance threshold set at *p* < 0.05.

### PPI network construction

We constructed a PPI network of the DEGs using the STRING database (https://string-db.org/) [18] to investigate targets’ interactions and involvement in the pathogenesis of DFU. We utilized Cytoscape 3.8.0 for the analysis and visualization of the results. To assess the importance of each node in the PPI network, we employed nine algorithms available in the CytoHubba plugin [betweenness, radiality, maximum neighborhood component, edge percolated component, density of the maximum neighborhood component, and maximal clique centrality (MCC)], degree, clustering coefficient, and closeness). These algorithms provide measures of centrality and connectivity, enabling the identification of key genes and proteins in the network.

### TF-miRNA hub EP-DEG network construction

We searched for TFs and miRNAs associated with the hub EP-DEGs on the NetworkAnalyst website (https://www.networkanalyst.ca/) [19]. We obtained information on TFs related to hub EP-DEGs from the ENCODE (https://www.encodeproject.org/chip-seq/transcription_factor/) [20], JASPAR (https://jaspar.genereg.net/) [21], and ChEA databases [22], and that on miRNAs associated with hub EP-DEGs from the TarBase and miRTarBase databases [23]. Information on the co-regulatory network involving both TFs and miRNAs was retrieved from the RegNetwork database (http://www.regnetworkweb.org/) [24]. We performed a comparative analysis of these data to identify key TFs and miRNAs. To visualize and analyze the resulting interaction networks, we used the Cytoscape 3.8.0 software.

### Patient sample collection

Twenty-four pairs of full-thickness skin tissue samples (12 each from patients with DFUs and healthy controls) were collected for the validation of candidate genes. The recruitment of participants for this study commenced on January 1, 2023, and concluded on May 31, 2023. DFU was diagnosed according to the 2020 Multidisciplinary Guidelines for the Prevention and Treatment of Diabetic Foot Disease [25]. The control group consisted of patients undergoing surgery for acute wounds or orthopedic conditions unrelated to diabetes. The selection criteria for both groups were age 50–80 years and no history of sepsis, autoimmune disease, malignancy, or other chronic disease; male and female patients were eligible. DFU severity was classified using the Wagner and Texas scales [26]. Basic information and health status histories were documented. All participants were thoroughly informed about the aims, methods, and potential risks and benefits of the study, and provided written informed consent, or verbal consent with third-party witnessing and documentation when necessary. The study was conducted in strict accordance with the Declaration of Helsinki (as revised in 2013), and with the approval of the Ethics Committee of Hubei Hospital of Traditional Chinese Medicine, affiliated with Hubei University of Chinese Medicine (no. HBZY2016-C64-01).

### Real-time fluorescence-based quantitative polymerase chain reaction

In a sterile operating room, the collected skin tissue specimens were quickly frozen in liquid nitrogen and then stored at –80°C until analysis. Total RNA was extracted from the samples by using the TRIpure total RNA extraction reagent kit (ELK Biotechnology, Wuhan, China) according to the manufacturer’s instructions. First-strand cDNA synthesis was performed using an EntiLink™ kit (EQ031; ELK Biotechnology). Fluorescence-based real-time quantitative polymerase chain reaction (RT-qPCR) was carried out using a QuantStudio 6 Flex System device (Life Technologies, Carlsbad, CA, USA). Each sample was run in triplicate using the EnTurbo™ SYBR Green PCR SuperMix kit (EQ001; ELK Biotechnology). The relative expression levels of the target genes were calculated by comparison with the reference genes using the comparative cycle threshold (Ct) method. The delta Ct value for each target and reference gene was obtained as ΔCt = Ct_target–Ct_reference. The relative expression level of the target gene relative to the reference gene in each sample was then obtained as ΔΔCt = ΔCt_sample–ΔCt_control. Finally, the relative expression was calculated as 2^(-ΔΔCt). The primer sequences used in the RT-qPCR analysis are provided in S2 Table.

### Statistical analysis

All statistical analyses were performed using R (version 4.2.1). The following R packages were used: Biobase (2.58.0), GEOquery (2.66.0), limma (3.54.0), DESeq (21.38.3), ggplot2 (3.3.6), ComplexHeatmap (2.13.1), clusterProfiler (4.4.4), and stats (4.2.1). For numerical variables, statistical methods were applied based on the normality and homogeneity of variances. The *t* test was used for data that met the assumptions of normal distribution and homogeneity of variance. Welch’s *t* test was used for data that met the assumption of normal distribution but not that of homogeneity of variance. The Wilcoxon test was used for data that did not meet the assumption of normal distribution. For categorical variables, group comparisons were conducted using Fisher’s exact test.

## Results

### DEG acquisition and expression

**Fig 2:**
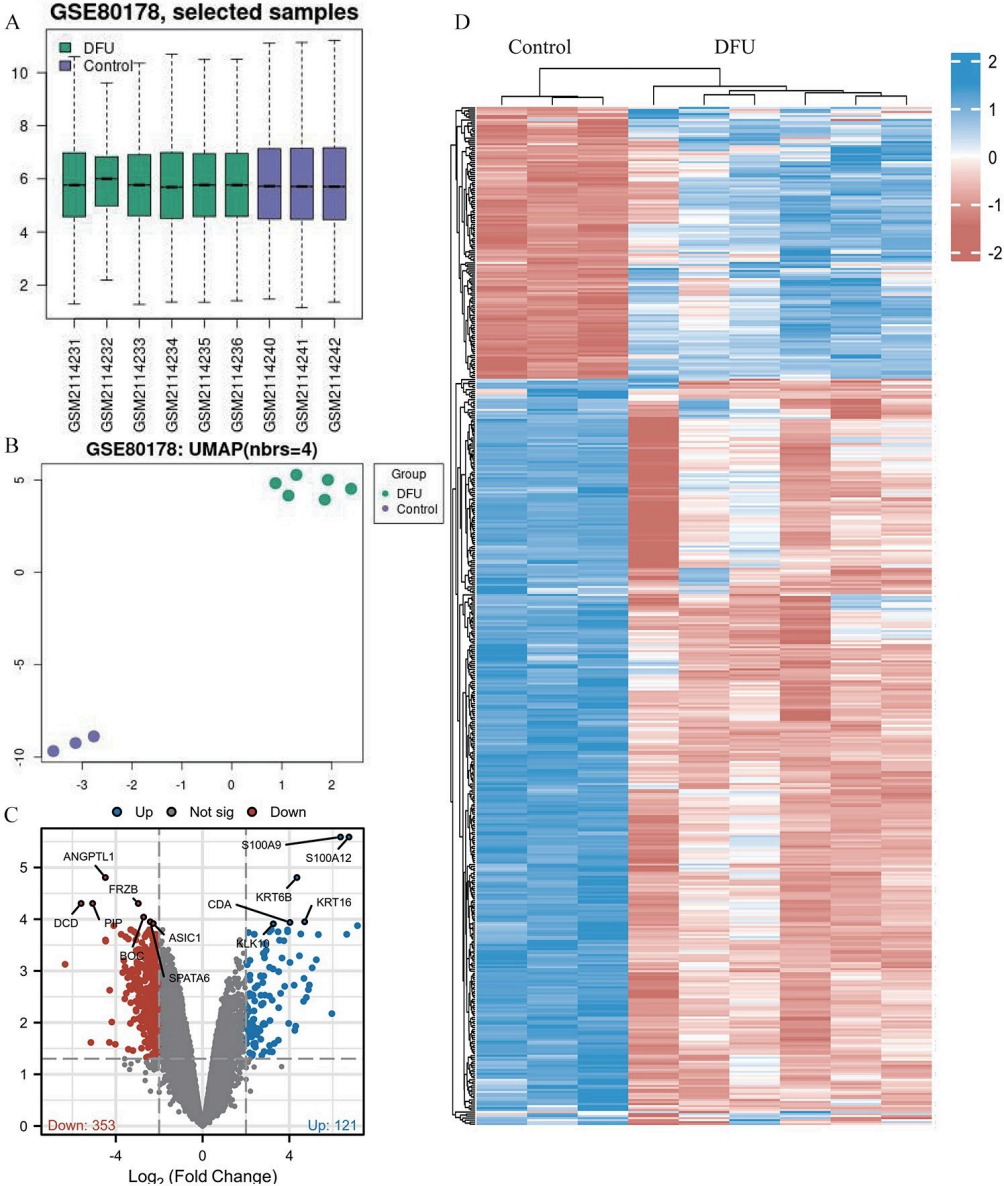
Analysis of DEGs in GSE80178 dataset. (A) Box plots showing gene expression distribution among samples in the DFU and control groups. The x-axis represents different samples, and the y-axis represents gene expression levels, displaying the median, quartiles, and outliers. (B) UMAP plot illustrating the two-dimensional projection of the dataset samples. The UMAP plot shows distinct clustering of DFU samples (green) and control samples (purple), indicating clear separation and comparability between the two groups based on their gene expression profiles. (C) Volcano plot of differential gene expression analysis. The x-axis represents log_2_ fold change, and the y-axis represents adjusted p-values. Red dots represent downregulated genes, and blue dots represent upregulated genes, with thresholds set at |log_2_FoldChange|>2 and p.adj <0.05. (D) Heatmap depicting the expression patterns of the identified 474 DEGs between DFU and control groups. Rows represent genes, and columns represent samples. The color scale indicates gene expression levels, with blue representing low expression and red representing high expression.

The box plot of the raw data obtained from the GEO2R online analysis (GSE80178; median, quartiles, and outliers) showed no significant difference in gene expression (Fig 2A). The uniform manifold approximation and projection (UMAP) plot showed distinct clustering of the DFU and control groups, reflecting clear separation based on gene expression profiles (Fig 2B). We identified a total of 474 DEGs (121 upregulated and 353 down-regulated) based on the set threshold (Fig 2C; S3 Table). The most significantly upregulated genes were S100A9, S100A12, KRT6B, KRT16, CDA, and KLK10. The most significantly down-regulated genes were ANGPTL1, FRZB, PIP, DCD, BOC, SPATA6, and ASIC1. The heatmap of DEGs showed distinct expression patterns for the DFU and control groups (Fig 2D).

### EP-DEG acquisition and expression

**Fig 3:**
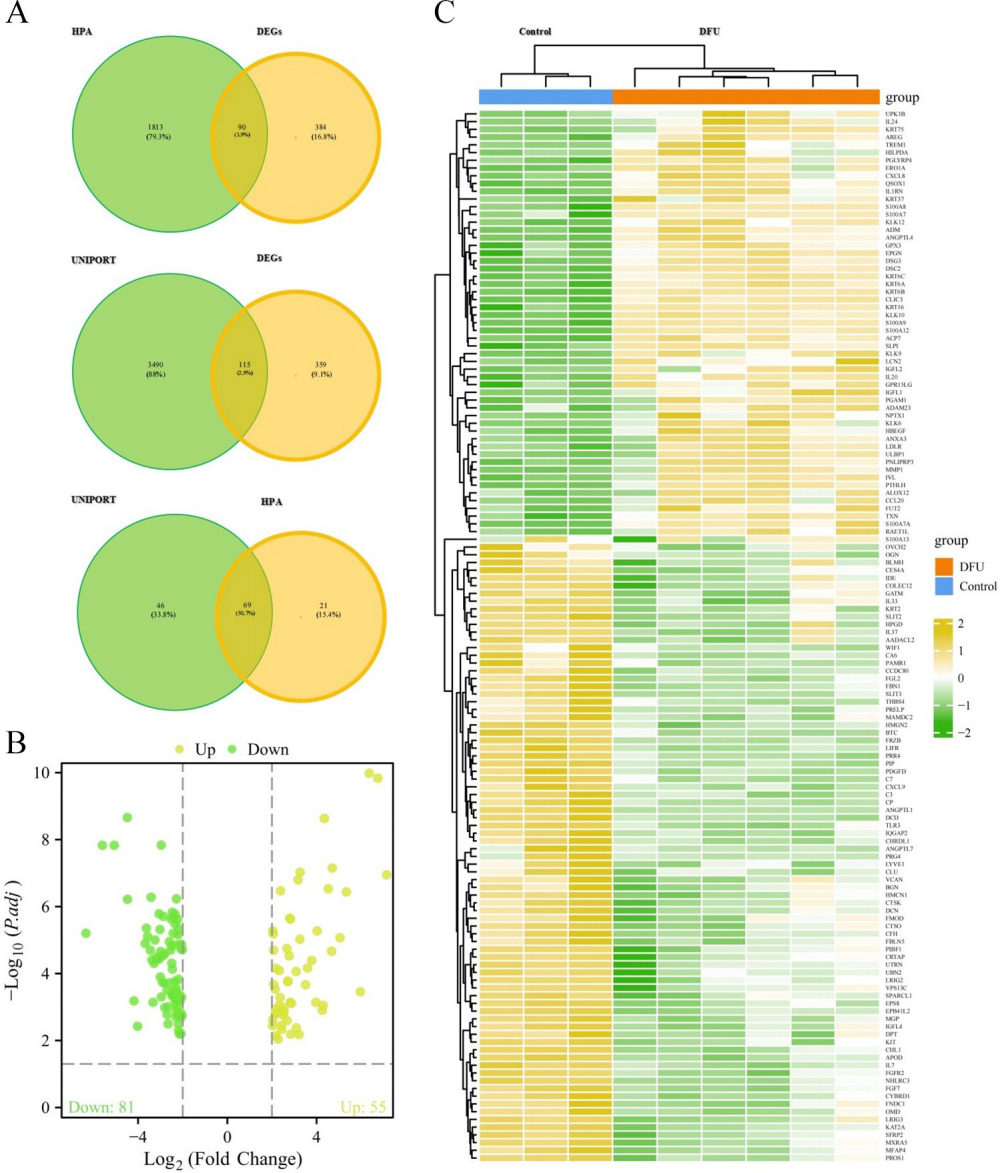
EP-DEG acquisition and expression. (A) Venn diagram showing the intersection of differentially expressed genes (DEGs) with the annotated extracellular protein (EP) databases: The intersection of DEGs with the extracellular protein annotation list downloaded from the Human Protein Atlas (HPA) database resulted in 90 EP-DEGs. The intersection of DEGs with the extracellular protein annotation list downloaded from the UniProt database resulted in 115 EP-DEGs. By merging the results of both intersections, a total of 136 EP-DEGs were obtained. (B, C) Volcano plot and heatmap illustrating the differential expression of EP-DEGs.

We obtained a gene list for 3,605 EPs from GO0005615 using UniProt. The examination of intersections of these EPs with the DEGs led to the identification of 115 differentially expressed proteins. Additionally, we retrieved a list of 1,903 EPs from the HPA database, and the examination of intersections with DEGs led to the identification of 90 differentially expressed proteins. After the removal of duplicate proteins, we obtained a total of 136 differentially expressed EPs (55 upregulated and 81 down-regulated; Fig 3A and 3B; S4 Table). The top three significantly upregulated genes were S100A9, S100A12, and KRT6B, and the top three significantly down-regulated genes were ANGPTL1, FRZB, and PIP. Fig 3C is a heatmap of the intergroup comparison of the EP-DEGs.

### Results of GO and KEGG analysis of EP-DEGs

**Fig 4:**
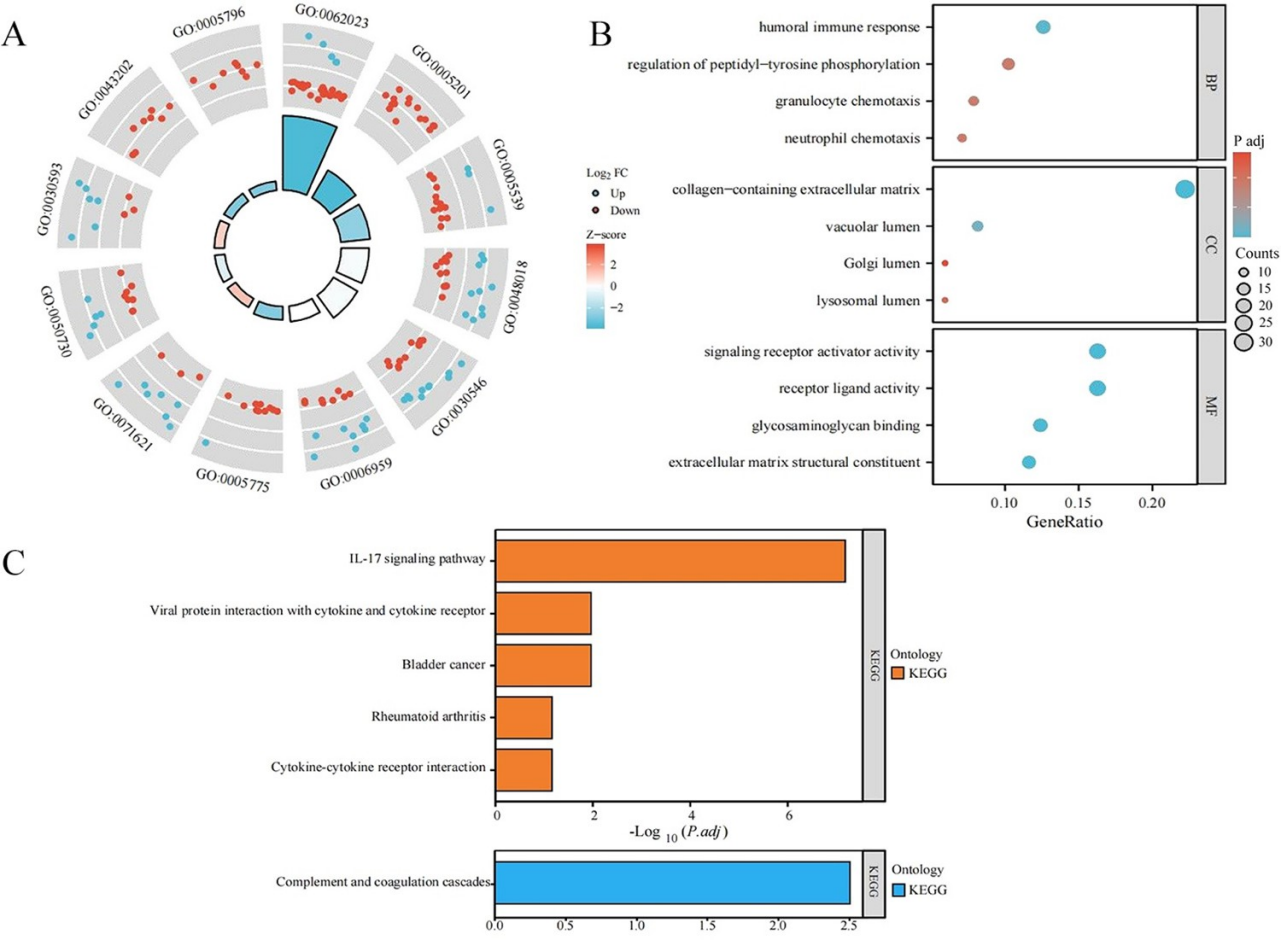
GO and KEGG analysis of EP-DEGs. (A) Circular plot showing the combination of Gene Ontology (GO) and KEGG pathway enrichment analysis results for EP-DEGs with fold change (FC) values. (B) Results of GO functional enrichment analysis for EP-DEGs. (C) Results of KEGG pathway enrichment analysis for EP-DEGs. The pathways predominantly enriched with upregulated genes are represented in red, while the pathways predominantly enriched with downregulated genes are represented in blue.

The GO analysis showed that the 136 EP-DEGs were enriched primarily in the following BPs: humoral immune response, granulocyte chemotaxis, regulation of peptidyl-tyrosine phosphorylation, and neutrophil chemotaxis. The EP-DEGs were enriched in MFs related to the collagen-containing ECM, vacuolar lumen, lysosomal lumen, and Golgi lumen. Among CCs, they were related primarily to ECM structural constituents, glycosaminoglycan (GAG) binding, receptor-ligand activity, and signaling receptor activator activity (Fig 4A and 4B; S5 Table). The KEGG pathway enrichment analysis showed that the 55 upregulated genes were related primarily to the interleukin (IL)-17 signaling pathway, bladder cancer, viral protein interaction with cytokines and cytokine receptors, cytokine–cytokine receptor interaction, and rheumatoid arthritis. The 81 down-regulated genes were associated mainly with the complement and coagulation cascades signaling pathway (Fig 4C).

### PPI network construction and hub EP-DEG screening

**Fig 5:**
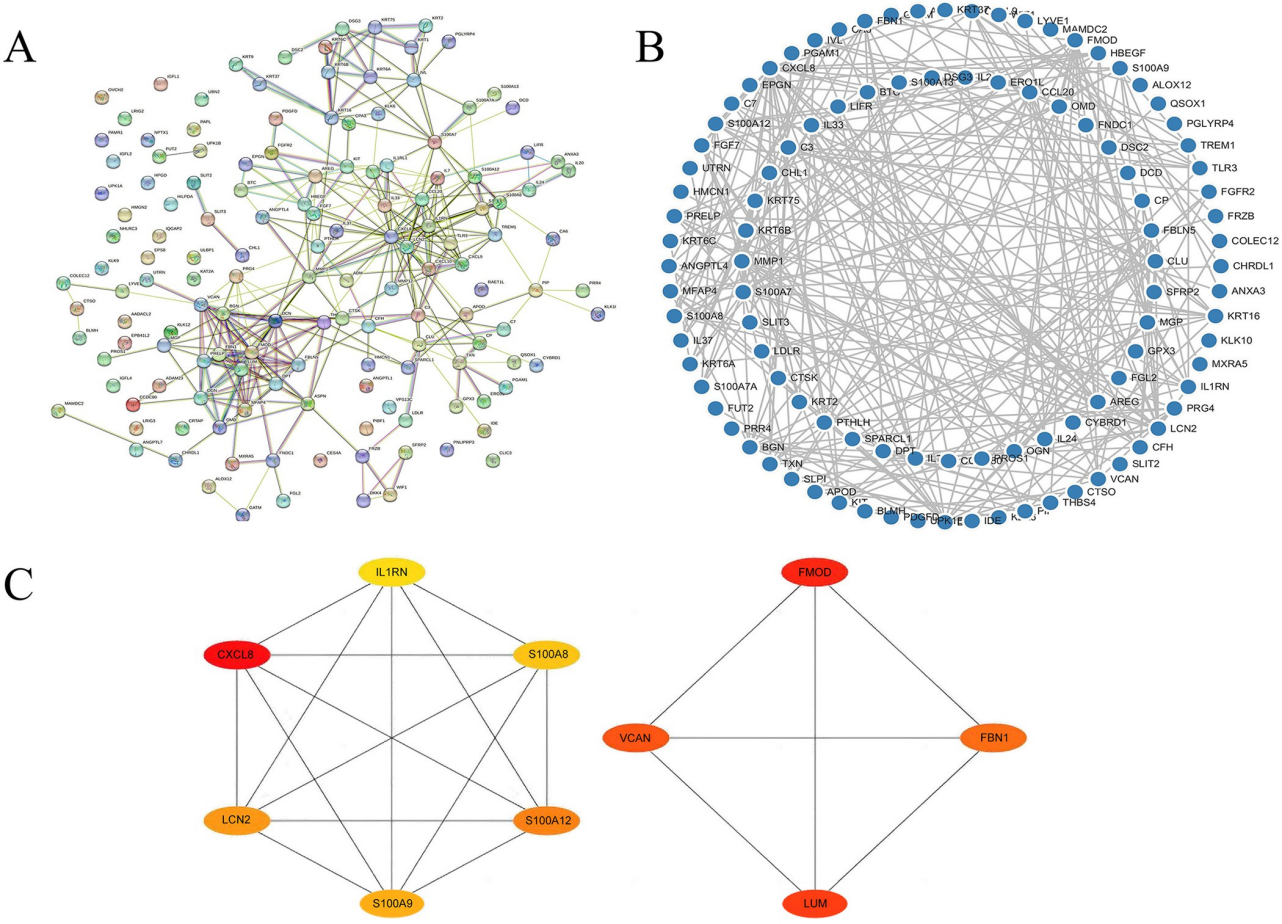
PPI network and identification of hub EP-DEGs. (A) PPI network graph of EP-DEGs shown by the STRING database. (B) Visualization analysis results of the PPI network graph using Cytoscape software. (C) Top ten hub EP-DEGs selected using the MCC algorithm.

The PPI network contained 136 EP-DEGs serving as nodes (Fig 5A; S6 Table). The refined network is shown in Fig 5B. By applying the MCC algorithm, we identified 10 hub genes: FMOD, LUM, VCAN, FBN1, IL1RN, S100A8, S100A12, S100A9, LCN2, and CXCL8 (Fig 5C).

### Patient characteristics and RT-qPCR verification of hub EP-DEGs

The DFU group comprised seven men and five women with an average age of 65.83 ± 11.97 years. The control group comprised six men and six women with an average age of 64.25 ± 11.06 years. All participants were of Chinese nationality. The erythrocyte sedimentation rate and leukocyte index did not differ between groups. The average glycated hemoglobin levels on admission were 8.28 ± 1.10 in the DFU group and 4.38 ± 0.34 in the control group (*p* < 0.001).

The baseline characteristics of the patients, including the blood pressure, history of heart attack, kidney disease, and diabetes management method (insulin or oral medication), are presented in S7 Table. The DFU group had an average fasting blood glucose level of 150 ± 25 mg/dL and a postprandial blood glucose level of 200 ± 30 mg/dL; these levels were 90 ± 10 and 120 ± 15 mg/dL, respectively, in the control group (both *p* < 0.001).

**Fig 6:**
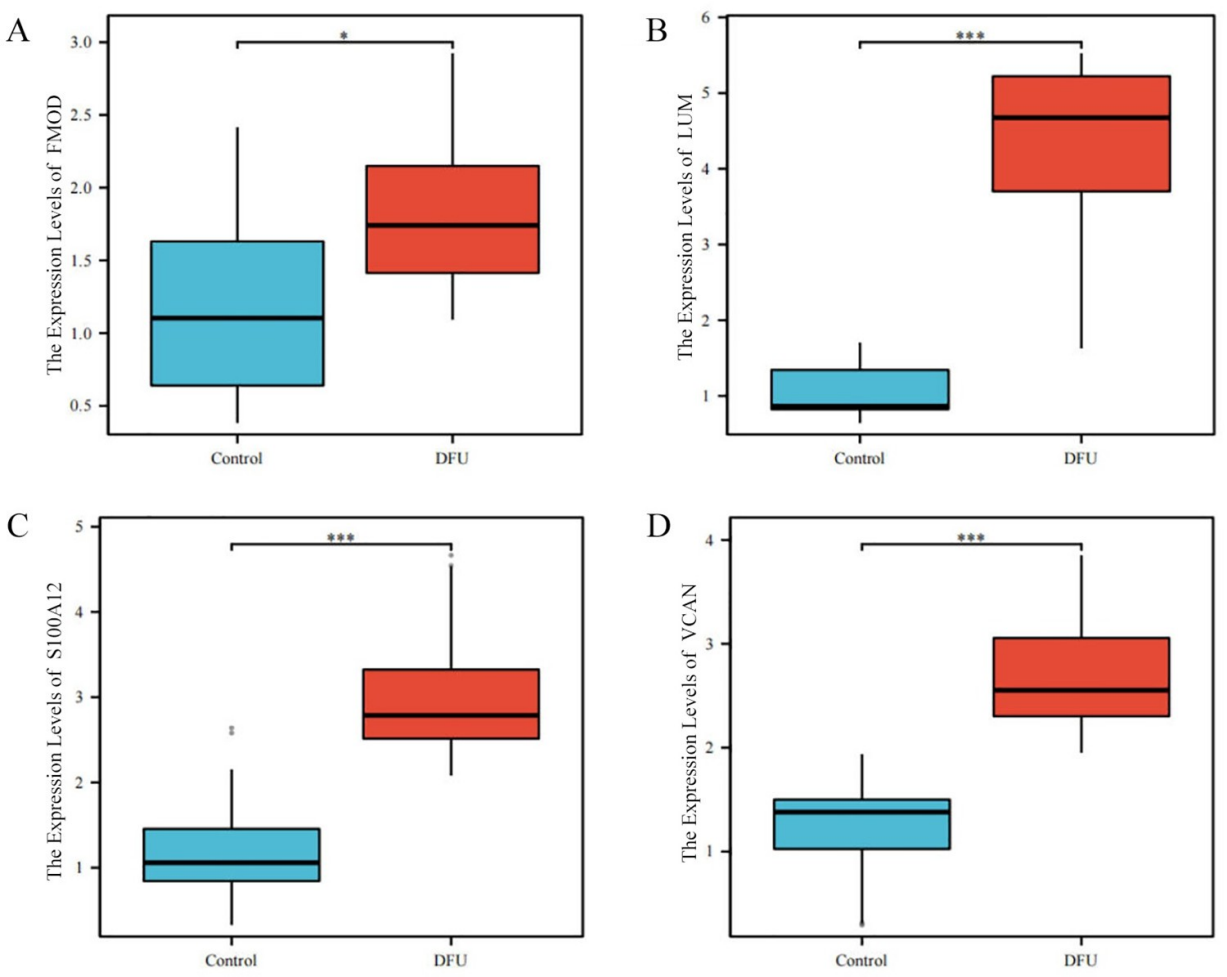
RT-qPCR verification of hub EP-DEGs. (A-D) Box plots showing the expression levels of FMOD (A), LUM (B), S100A12 (C), and VCAN (D) in the DFU group compared to the control group. (* p < 0.05, *** p < 0.001).

The RT-qPCR analysis revealed differential expression of the FOMD, LUM, S100A12, and VCAN genes between groups, with all being upregulated in the DFU group (Fig 6; S8 Table). No significant between-group difference in the expression of the remaining six genes was observed.

### Construction of the TF-miRNA co-regulatory network for hub EP-DEGs

**Fig 7:**
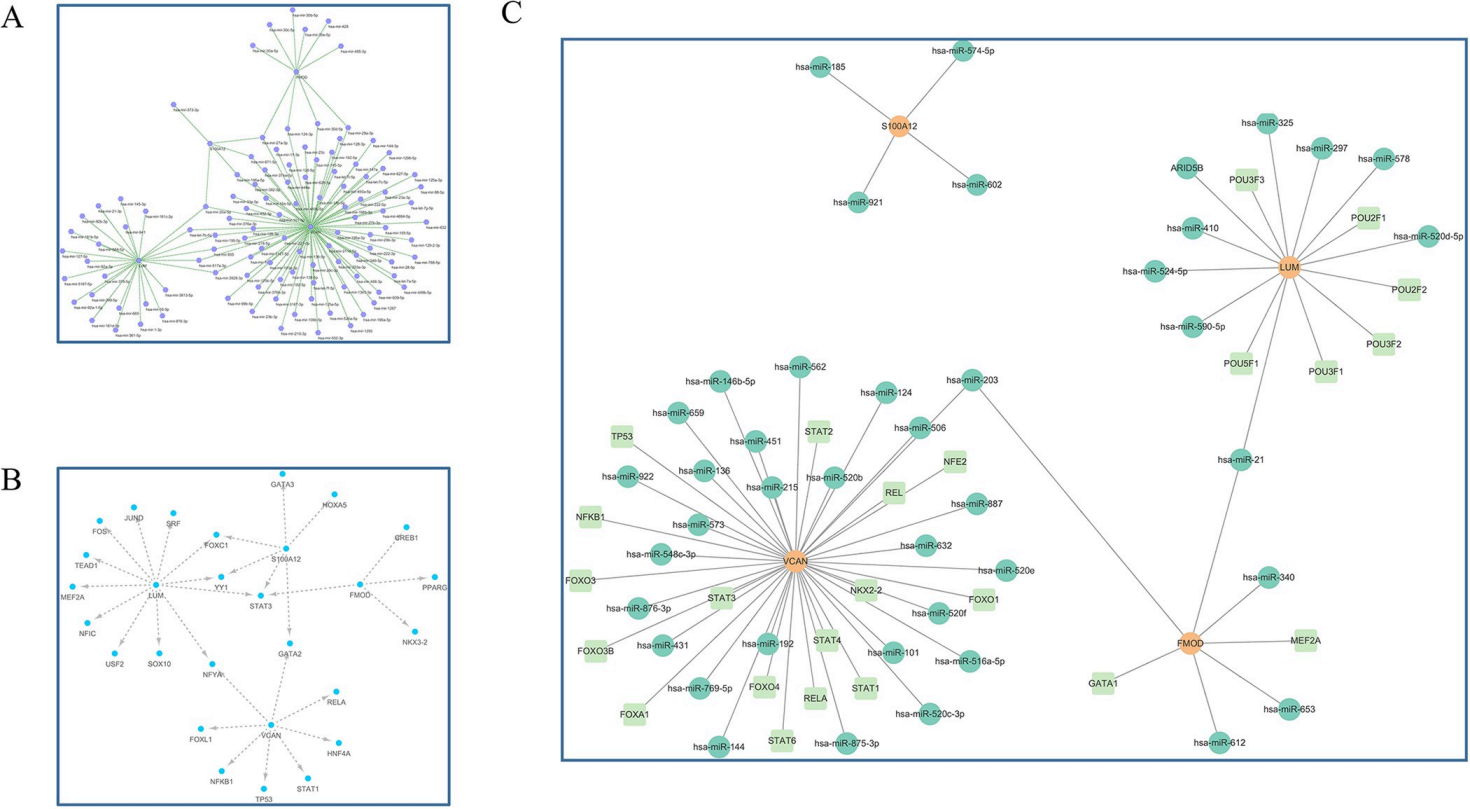
Construction of the TF-miRNA co-regulatory network for hub EP-DEGs. (A) Network diagram of miRNA-Hub DEGs predicted based on the TarBase database. (B) Network diagram of TF-Hub DEGs predicted based on the JASPAR database. (C) Co-regulatory network diagram of TF-miRNA-Hub genes predicted based on the RegNetwork database.

The examination of ChEA data revealed that the TF tripartite motif-containing 28 (TRIM28) interacts with three hub genes with highly significant differential expression: LUM, VCAN, and FMOD (S1 Fig). TarBase data revealed that hsa-mir-20a-5p interacts with LUM, VCAN, and S100A12 (Fig 7A). The JASPAR data analysis showed that the TF signal transducer and activator of transcription 3 (STAT3) interact with LUM, VCAN, and S100A12 (Fig 7B). In the co-regulatory TF-miRNA–hub gene network based on RegNetwork data, hsa-miR-21 and hsa-miR-203 were found to regulate the experimentally validated hub genes FMOD, LUM, and VCAN (Fig 7C). Thus, TRIM28 and STAT3 were identified as potential upstream and downstream regulatory factors, and hsa-mir-20a-5p, hsa-miR-21, and hsa-miR-203 were identified as potentially associated miRNAs.

## Discussion

DFUs, a serious complication of diabetes, result from a complex interplay of multiple factors, and their pathogenesis remains incompletely understood [27]. Emerging evidence highlights the critical role of EPs in the development of DFUs. These proteins, which include collagen, elastin, and matrix metalloproteinases, are integral components of the ECM and are essential for the maintenance of tissue integrity. They participate in key BPs such as the provision of structural tissue support, facilitation of cell-cell interactions, and mediation of signal transduction [28]. An understanding of the role of these EPs in DFU development can provide insights into the mechanisms by which ECM dysregulation contributes to ulcer formation and the impairment of wound healing in diabetic patients.

In this study, we conducted a comprehensive bioinformatics analysis of gene expression data from patients with DFU and healthy controls in the GSE80178 microarray dataset. We identified 474 DEGs, 121 of which were upregulated and 353 of which were down-regulated. By integrating data from the HPA and UniProt databases, which contain extensive human protein data, we identified 136 EP-DEGs. GO enrichment analysis of these EP-DEGs revealed their involvement in several critical BPs. Notably, these genes are implicated in the humoral immune response, a crucial aspect of the immune system involving antibody production and the activity of immune molecules in body fluids. The activation of this response suggests a link to the inflammation and immune dysregulation that occur in the pathogenesis of DFU [29]. Furthermore, the identification of links to processes such as granulocyte and neutrophil chemotaxis underscores the role of inflammatory cell migration and activation in DFU development. These findings indicate that inflammatory signals and tissue damage drive granulocyte chemotaxis and activation, exacerbating ulcer formation [30]. Additionally, the association with the regulation of peptidyl-tyrosine phosphorylation, a key mechanism in cellular signal transduction, is relevant to the inflammatory, proliferative, and tissue repair processes integral to DFU development. Abnormal phosphorylation may thus contribute to the chronic inflammation and impaired healing that characterize DFUs [31]. Among CCs, the association with collagen-containing ECM activity is noteworthy. The ECM is a supportive structure surrounding cells that contains abundant collagen proteins. In DFUs, abnormal ECM accumulation, remodeling, and degradation are associated with changes in tissue structure and impaired wound healing [32]. Our KEGG pathway analysis further revealed significant enrichment in the IL-17 signaling pathway, underscoring the roles of this pathway in the mediation of inflammatory responses and immune regulation in DFUs. Additionally, the involvement of complement and coagulation cascade pathways suggests that vascular complications, such as impaired blood flow and increased thrombosis, are critical factors in DFU pathology [33].

All of these cellular functions and signaling pathways are integral to the pathogenesis of DFUs. These findings underscore the multifactorial nature of DFUs, involving processes such as inflammation, immune regulation, cell migration, ECM remodeling, vascular function, and thrombosis. They provide valuable insights into the mechanisms underlying the DFU pathology and highlight potential targets for therapeutic intervention.

Our PPI network analysis identified 10 hub genes, 4 (FMOD, LUM, S100A12, and VCAN) of which showed significant differential expression in DFU samples. These proteins are integral to ECM organization and wound healing. Fibromodulin (FMOD) is an ECM protein from the small leucine-rich proteoglycan family, found predominantly in connective tissues. It plays a pivotal role in collagen fiber formation and regulation, influencing collagen fiber diameter, morphology, and mechanical properties [34]. This regulation is crucial for collagen fiber regeneration, tissue remodeling, and the reinforcement of the ECM’s mechanical characteristics. The role of FMOD in wound healing is well documented, and its derived peptide SLI-F06 shows potential for the promotion of wound closure and enhancement of myofibroblast function, making it a promising candidate for the treatment of chronic, non-healing diabetic wounds [35].

Similar to FMOD, the ECM component lumican (LUM) plays crucial roles in the regulation of collagen fiber and tissue repair. It increases the diameter and stability of collagen fibers, maintains the mechanical properties of the ECM, and participates in the repair of damaged tissues. LUM is involved in the promotion of cell migration and proliferation and the regulation of collagen fiber regeneration and remodeling [36]. It also exerts anti-inflammatory effects by inhibiting the release of inflammatory factors and regulating the activation state of inflammatory cells [37]. These properties suggest that LUM not only supports tissue integrity, but also modulates the inflammatory environment, thereby contributing to the resolution of inflammation and enhancement of healing process in DFUs.

Versican (VCAN), a member of the proteoglycan family, is composed of a core protein and multiple GAG chains. The core protein has G1–3 and GAGα domains [38]. The highly sulfated and methylated GAG component allows VCAN to interact with other ECM proteins, such as collagen and fibronectin, forming a complex network that provides essential tissue support and elasticity. In a study of non-specific immunostaining in diabetic skin, Loots et al. [37] found that VCAN deposition exceeded the normal healing time frame. Notably, VCAN was detected in high levels in the dermis and basement membrane in DFUs, and its upregulation persisted for more than 12 months [39]. This persistent and prolonged upregulation of VCAN aligns with our findings; it may disrupt normal ECM dynamics, contributing to the impaired healing observed in DFUs. These findings suggest that VCAN not only plays a structural role, but is also a potential diagnostic biomarker for DFU chronicity and severity.

S100A12, a calcium-binding protein from the S100 family, plays crucial roles in the regulation of inflammation and immune responses. Elevated S100A12 expression has been associated with various inflammatory conditions, including arthritis [40], inflammatory bowel disease [41], and atherosclerosis [42], making it a key indicator of inflammation. Within the S100 family, S100A12 shares structural and functional similarities with S100A8 and S100A9, which form heterodimers such as the S100A8/A9 complex (calprotectin) [43]. These complexes are instrumental in the modulation of inflammatory and immune responses. Significant upregulation of S100A8 and S100A9 in DFU wound exudates has been reported, but the role of S100A12 remained unexplored [44]. In this study, we identified significantly differential expression of S100A12 between patients with DFU and healthy controls, which was validated through the fluorescence-based RT-qPCR analysis of skin specimens. These findings not only align with, but also expand upon, previous research, underscoring the relevance of regulatory interactions within the S100 family to the pathogenesis of DFU. By elucidating the role of S100A12 in DFU, our study contributes to a more comprehensive understanding of the inflammatory mechanisms underlying this condition and identifies S100A12 as a potential biomarker and therapeutic target for DFU.

To elucidate the regulatory mechanisms underlying DFUs, we used NetworkAnalyst to characterize the TF-miRNA regulatory networks of the core DEGs validated by PCR. We identified TRIM28 and STAT3 as key upstream TFs and hsa-mir-20a-5p, hsa-miR-21, and hsa-miR-203 as significant downstream miRNAs regulating these DEGs. Although these findings provide a foundational understanding of regulatory interactions involved in the pathogenesis of DFUs, further mechanistic studies are required to validate and clarify these interactions. MiR-21 [45], miR-20a [46], and miR-203 [47] have been implicated in wound healing and DFU formation. For instance, Madhyastha et al. [48] demonstrated that miR-21 is crucial for fibroblast migration, a key event in the promotion of growth factor secretion and recruitment of other cell types to wound sites. Interestingly, miR-21 expression is increased in diabetic skin but decreased during diabetic wound healing, highlighting its dynamic role in the pathogenesis of DFU. Similarly, Li et al. [49] found that miR-20a facilitates wound healing by inhibiting semaphorin 7A expression in the nuclear factor-κB signaling pathway, thereby reducing inflammatory factor production and controlling inflammatory responses at wound sites. These insights underscore the complexity of the regulatory networks involving TFs and miRNAs that participate in the pathogenesis of DFU. A better understanding of these networks could lead to the identification of novel therapeutic targets for the improvement of DFU treatment and healing outcomes.

## Limitation

This study was conducted using data from online databases, which have inherent limitations. Additionally, the use of a single dataset may limit the generalizability of our findings. Future studies should integrate multiple datasets to provide a more robust analysis. Another main limitation of the study is the small skin tissue sample, which may limit the generalizability of our findings. However, we used rigorous inclusion criteria and advanced bioinformatics analyses to ensure reliability. Future studies should be conducted with larger samples to confirm and extend our findings. Additionally, transcriptomics analysis should be utilized to investigate the regulatory networks associated with the DEGs identified in this study. The adoption of these approaches will provide a more comprehensive understanding of the molecular basis of DFU pathogenesis and may facilitate the development of novel therapeutic strategies.

## Conclusion

In this study, we elucidated the roles of key EPs in DFUs, combining online database analyses with GO and KEGG pathway analysis to detail these proteins’ functions and regulatory mechanisms. We validated the differential expression of FMOD, LUM, S100A12, and VCAN in DFUs, thereby addressing critical gaps in our understanding of this condition. Additionally, we constructed a co-regulatory network involving the TFs TRIM28 and STAT3 and miRNAs hsa-mir-20a-5p, hsa-miR-21, and hsa-miR-203, offering new insights into gene regulation. This integrative approach not only advances our understanding of the molecular basis of DFU pathogenesis, but also provides potential targets for therapeutic intervention.

## Supporting information

S1 Fig Network diagram of TF-Hub DEGs predicted based on the CheA database. (PDF)

S1 Table Gene expression in the GSE80178 dataset. (XLSX)

S2 Table PCR primer sequence table. (DOCX)

S3 Table 474 DEGs. (XLSX)

S4 Table 136 EP-DEGs. (XLSX)

S5 Table The results of the enrichment analysis combining GO and KEGG. (DOCX)

S6 Table PPI network. (XLSX)

S7 Table Baseline characteristics of included patients. (DOCX)

S8 Table RT-qPCR analysis results for FOMD, LUM, S100A12, and VCAN genes. (XLSX)
