# Supplementary figures and images for: Key extracellular proteins and TF-miRNA co-regulatory network in diabetic foot ulcer: Bioinformatics and experimental insights
Source: PLoS One. 2024 Jul 22;19(7):e0307205. doi: 10.1371/journal.pone.0307205 (PMC11262672; doi:10.1371/journal.pone.0307205)

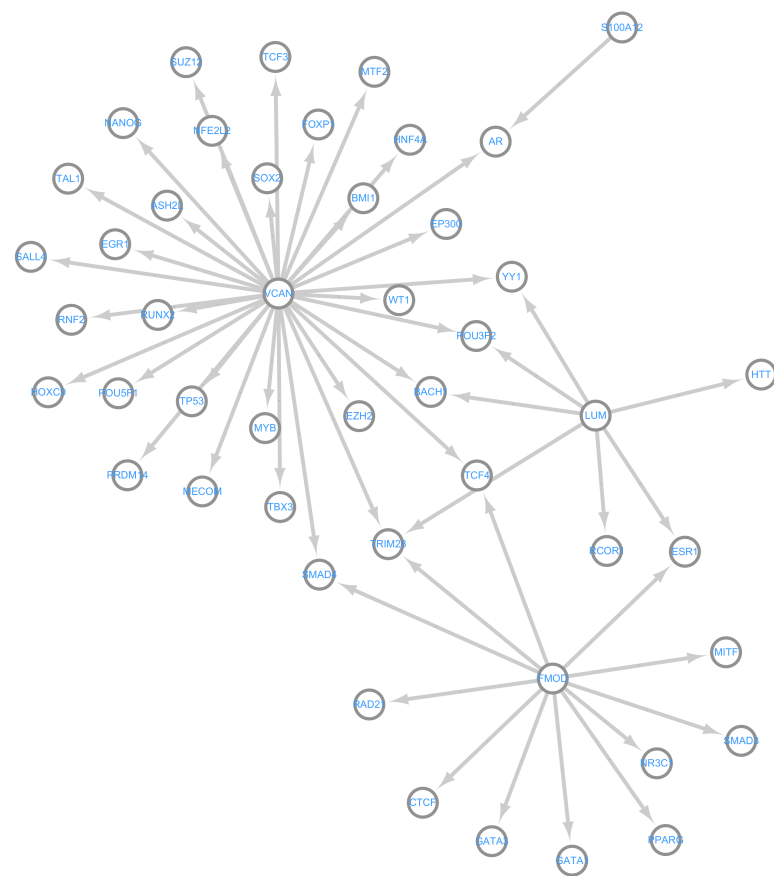

Supplement: S1 Fig — (PDF) [file pone.0307205.s001.PDF]
