# Supplementary material for: Key extracellular proteins and TF-miRNA co-regulatory network in diabetic foot ulcer: Bioinformatics and experimental insights
Source: PLoS One. 2024 Jul 22;19(7):e0307205. doi: 10.1371/journal.pone.0307205 (PMC11262672; doi:10.1371/journal.pone.0307205)
Supplement: S2 Table — (DOCX) [file pone.0307205.s003.DOCX]

S2 Table. PCR primer sequence table

| **Primer name** | **Base sequence (5 '-3')** | | **Product length** |
| --- | --- | --- | --- |
| β-actin | sense | GTCCACCGCAAATGCTTCTA | 190 |
|  | antisense | TGCTGTCACCTTCACCGTTC |  |
| FMOD | sense | CCTGAGTTATAACCACCTTCGG | 222 |
|  | antisense | TTGTAGGAGAGGTCTAGCTCAAGG |  |
| S100A12 | sense | GGTTAACATTAGGCTGGGAAGAT | 190 |
|  | antisense | TTTCATCAATGACAGCTTTATCTTT |  |
| LUM | sense | CTATCTTATGGTACAGCGGCCT | 160 |
|  | antisense | GAGGCTGCTATCACACTCTAAGG |  |
| VCAN | sense | CCTTAATAGTAACCCATGCGCT | 135 |
|  | antisense | TGGTGTTGTAACTGGGTGGC |  |
