# Supplementary material for: Key extracellular proteins and TF-miRNA co-regulatory network in diabetic foot ulcer: Bioinformatics and experimental insights
Source: PLoS One. 2024 Jul 22;19(7):e0307205. doi: 10.1371/journal.pone.0307205 (PMC11262672; doi:10.1371/journal.pone.0307205)
Supplement: S5 Table — (DOCX) [file pone.0307205.s006.DOCX]

S5 Table. The results of the enrichment analysis combining GO and KEGG

| Ontology | ID | Description | GeneRatio | BgRatio | pvalue | p.adjust | zscore |
| --- | --- | --- | --- | --- | --- | --- | --- |
| BP | GO:0006959 | humoral immune response | 16/127 | 317/18800 | 4.42e-10 | 9.8e-07 | 0 |
| BP | GO:0071621 | granulocyte chemotaxis | 10/127 | 128/18800 | 1.6e-08 | 1.77e-05 | 1.2649 |
| BP | GO:0050730 | regulation of peptidyl-tyrosine phosphorylation | 13/127 | 261/18800 | 2.46e-08 | 1.82e-05 | -0.83205 |
| BP | GO:0030593 | neutrophil chemotaxis | 9/127 | 106/18800 | 4.16e-08 | 1.85e-05 | 1 |
| BP | GO:0050900 | leukocyte migration | 15/127 | 384/18800 | 5e-08 | 1.85e-05 | -0.2582 |
| CC | GO:0062023 | collagen-containing extracellular matrix | 30/135 | 429/19594 | 6.77e-22 | 1.26e-19 | -3.6515 |
| CC | GO:0005775 | vacuolar lumen | 11/135 | 174/19594 | 3.41e-08 | 3.17e-06 | -2.7136 |
| CC | GO:0043202 | lysosomal lumen | 8/135 | 97/19594 | 3.56e-07 | 2.21e-05 | -2.8284 |
| CC | GO:0005796 | Golgi lumen | 8/135 | 104/19594 | 6.09e-07 | 2.83e-05 | -2.8284 |
| CC | GO:0034774 | secretory granule lumen | 12/135 | 322/19594 | 2.41e-06 | 7.49e-05 | 1.1547 |
| MF | GO:0005201 | extracellular matrix structural constituent | 15/129 | 172/18410 | 1.16e-12 | 2.77e-10 | -3.873 |
| MF | GO:0005539 | glycosaminoglycan binding | 16/129 | 234/18410 | 8.29e-12 | 9.9e-10 | -2.5 |
| MF | GO:0048018 | receptor ligand activity | 21/129 | 489/18410 | 2.74e-11 | 2.14e-09 | -0.21822 |
| MF | GO:0030546 | signaling receptor activator activity | 21/129 | 496/18410 | 3.57e-11 | 2.14e-09 | -0.21822 |
| MF | GO:0030021 | extracellular matrix structural constituent conferring compression resistance | 7/129 | 22/18410 | 1.1e-10 | 5.26e-09 | -2.6458 |
| Ontology: Category of the function or pathway, including BP (Biological Process), CC (Cellular Component), MF (Molecular Function), and KEGG (Kyoto Encyclopedia of Genes and Genomes).  ID: Identifier number corresponding to the specific function or pathway.  Description: Name of the corresponding function or pathway.  GeneRatio: Ratio of the intersection of the input molecules (after ID conversion) with the total number of molecules annotated in the corresponding ID entry to the intersection of the input molecules (after ID conversion) with the total number of molecules annotated with functional information in the database (separate for BP, CC, MF, and KEGG).  BgRatio: Ratio of the total number of molecules in the corresponding ID entry to the intersection of the total number of molecules annotated with functional information in the database (separate for BP, CC, MF, and KEGG).  p-value: P-value from the hypergeometric distribution test.  p.adjust: Adjusted p-value obtained through p-value correction methods.  z-score: Standard score indicating the degree of deviation from the mean in a standard normal distribution. | | | | | | | |
