# Supplementary material for: Key extracellular proteins and TF-miRNA co-regulatory network in diabetic foot ulcer: Bioinformatics and experimental insights
Source: PLoS One. 2024 Jul 22;19(7):e0307205. doi: 10.1371/journal.pone.0307205 (PMC11262672; doi:10.1371/journal.pone.0307205)
Supplement: S7 Table — (DOCX) [file pone.0307205.s008.docx]

# S7 Table. Baseline characteristics of included patients

| Characteristics | DFU (n=12) | Control (n=12) | P value |
| --- | --- | --- | --- |
| Gender n (%) |  |  | 1.000 |
| Female | 5 (41.7%) | 6 (50%) |  |
| Male | 7 (58.3%) | 6 (50%) |  |
| Age mean ± sd | 65.833 ± 11.968 | 64.25 ± 11.063 | 0.740 |
| Nationality | Chinese (100%) | Chinese (100%) | - |
| HbA1C mean ± sd | 8.275 ± 1.0997 | 4.375 ± 0.33609 | < 0.001 |
| FBG mean ± sd (mg/dL) | 150 ± 25 | 90 ± 10 | < 0.001 |
| PBG mean ± sd (mg/dL) | 200 ± 30 | 120 ± 15 | < 0.001 |
| Blood Pressure (mean ± sd) | 140/90 ± 15/10 | 120/80 ± 10/5 | 0.020 |
| History of Heart Attacks n (%) | 3 (25%) | 1 (8.3%) | 0.250 |
| Kidney Disease n (%) | 2 (16.7%) | 0 (0%) | 0.140 |
| Diabetes Management |  |  |  |
| Insulin | 6 (50%) | 0 (0%) | < 0.001 |
| Oral Medication | 6 (50%) | 0 (0%) | < 0.001 |
| ESR median (IQR) | 42 (26.75 - 66) | 39 (28.5 - 50) | 0.795 |
| WBC mean ± sd | 8.045 ± 3.4816 | 8.0367 ± 2.6492 | 0.995 |
| Wagner Classification n (%) |  |  | < 0.001 |
| Grade 2 | 2 (16.7%) | 0 (0%) |  |
| Grade 4 | 6 (50%) | 0 (0%) |  |
| Grade 5 | 4 (33.3%) | 0 (0%) |  |
| Texas Classification n (%) |  |  | < 0.001 |
| D1 | 2 (16.7%) | 0 (0%) |  |
| D2 | 5 (41.7%) | 0 (0%) |  |
| D3 | 5 (41.7%) | 0 (0%) |  |

*Note: DFU = Diabetic Foot Ulcer, FBG = Fasting Blood Glucose, PBG = Postprandial Blood Glucose, HbA1C = Glycated Hemoglobin, ESR = Erythrocyte Sedimentation Rate, WBC = White Blood Cell count.*
